# Sensory Experience Differentially Modulates the mRNA Expression of the Polysialyltransferases ST8SiaII and ST8SiaIV in Postnatal Mouse Visual Cortex

**DOI:** 10.1371/journal.pone.0024874

**Published:** 2011-09-21

**Authors:** Marie-Claude Bélanger, Graziella Di Cristo

**Affiliations:** CHU Sainte-Justine and Université de Montréal, Montreal, Quebec, Canada; The Research Center of Neurobiology-Neurophysiology of Marseille, France

## Abstract

Polysialic acid (PSA) is a unique carbohydrate composed of a linear homopolymer of α-2,8 linked sialic acid, and is mainly attached to the fifth immunoglobulin-like domain of the neural cell adhesion molecule (NCAM) in vertebrate neural system. In the brain, PSA is exclusively synthesized by the two polysialyltransferases ST8SiaII (also known as STX) and ST8SiaIV (also known as PST). By modulating adhesive property of NCAM, PSA plays a critical role in several neural development processes such as cell migration, neurite outgrowth, axon pathfinding, synaptogenesis and activity-dependent plasticity. The expression of PSA is temporally and spatially regulated during neural development and a tight regulation of PSA expression is essential to its biological function. In mouse visual cortex, PSA is downregulated following eye opening and its decrease allows the maturation of GABAergic synapses and the opening of the critical period for ocular dominance plasticity. Relatively little is known about how PSA levels are regulated by sensory experience and neuronal activity. Here, we demonstrate that while both ST8SiaII and ST8SiaIV mRNA levels decrease around the time of eye opening in mouse visual cortex, only ST8SiaII mRNA level reduction is regulated by sensory experience. Using an organotypic culture system from mouse visual cortex, we further show that ST8SiaII gene expression is regulated by spiking activity and NMDA-mediated excitation. Further, we show that both ST8SiaII and ST8SiaIV mRNA levels are positively regulated by PKC-mediated signaling. Therefore, sensory experience-dependent ST8SiaII gene expression regulates PSA levels in postnatal visual cortex, thus acting as molecular link between visual activity and PSA expression.

## Introduction

Polysialic acid (PSA) moiety is a long, linear homopolymer of α-2,8-linked sialic acid attached almost exclusively to the neural cell adhesion molecule (NCAM) in vertebrates [1]. PSA modulates cell adhesion and signal transduction events mediated by NCAM and other adhesion molecules by virtue of its polyanionic nature and large hydrated volume [2].

In the developing nervous system, PSA has been shown to play a role in neuronal migration [3], axonal fasciculation, branching, and targeting in the peripheral and central nervous system [4], [5], [6], synaptogenesis [7], [8] and activity-dependent plasticity [9], [10]. In addition, the persistent expression of PSA in certain regions of the adult nervous system, including but not only the hippocampus, the olfactory bulb and the hypothalamus, is correlated with the maintenance of neurogenesis and circuit remodelling [11], [12].

As a widespread and general modulator of cell interaction, PSA in itself is unlikely to provide a specific signal for cell interactions [13]. Instead, its expression may represent a regulated permissive signal for allowing optimal levels of interactions between cell-cell or cell-extracellular matrix, which may either promote or inhibit specific morphogenic events at the appropriate time and with the appropriate order. In this context, a tight regulation of PSA expression appears to be essential to its biological function. A key issue is how the expression of PSA is regulated as a part of physiological process in the brain.

A recent study showed that PSA expression is downregulated in visual cortex after eye opening and its decline is dependent on sensory experience [8]. Premature removal of PSA induced early maturation of GABAergic innervation and onset of critical period for ocular dominance plasticity [8]. Critical periods represent heightened epochs of brain plasticity, during which experience can produce permanent, large-scale changes in neuronal circuits [14]. Experience-dependent refinement of neural circuits has been described in many regions within the central nervous system, suggesting it is a fundamental mechanism for normal vertebrate CNS development. By regulating the timing of the onset of critical periods, PSA expression may influence how experience shapes brain wiring during early life and adolescence. The cellular mechanisms that couple sensory experience and PSA expression in the developing brain are unknown.

NCAM is polysialylated by two polysialyltransferases ST8SiaII (also known as STX) [15] and ST8SiaIV (also known as PST) [16]. Studies of ST8SiaII and ST8SiaIV knockout mice have revealed specific and distinct deficits in synaptic connectivity and plasticity, and learning and memory process reinforcing their role in synapse formation and neural circuits function [17], [18], [19]. The ST8SiaII gene is strongly expressed in the fetal and neonatal brain, whereas ST8SiaIV gene expression predominates in the mature brain [20], but their individual roles *in vivo* are still not fully understood.

Here, we examine the role of sensory experience and neuronal activity in the regulation of ST8SiaII and ST8SiaIV gene expression in the postnatal brain. We showed that ST8SiaII and ST8SiaIV mRNA levels were down-regulated around the second postnatal week in mouse visual cortex, paralleling the decrease in PSA expression levels. The decline in ST8SiaII, but not ST8SiaIV mRNA levels, were dependent on visual experience *in vivo*. Consistent with these results, we further showed that neuronal activity levels, and in particular NMDA activation, regulate ST8SiaII but not ST8SiaIV expression, *in vitro*. Conversely, PKC positively regulated both ST8SiaIV and ST8SiaII expression. Altogether, our data suggest that sensory experience-dependent ST8SiaII expression regulates PSA levels in postnatal visual cortex, therefore defining a molecular link between visual activity and PSA expression.

## Results

**Figure 1:**
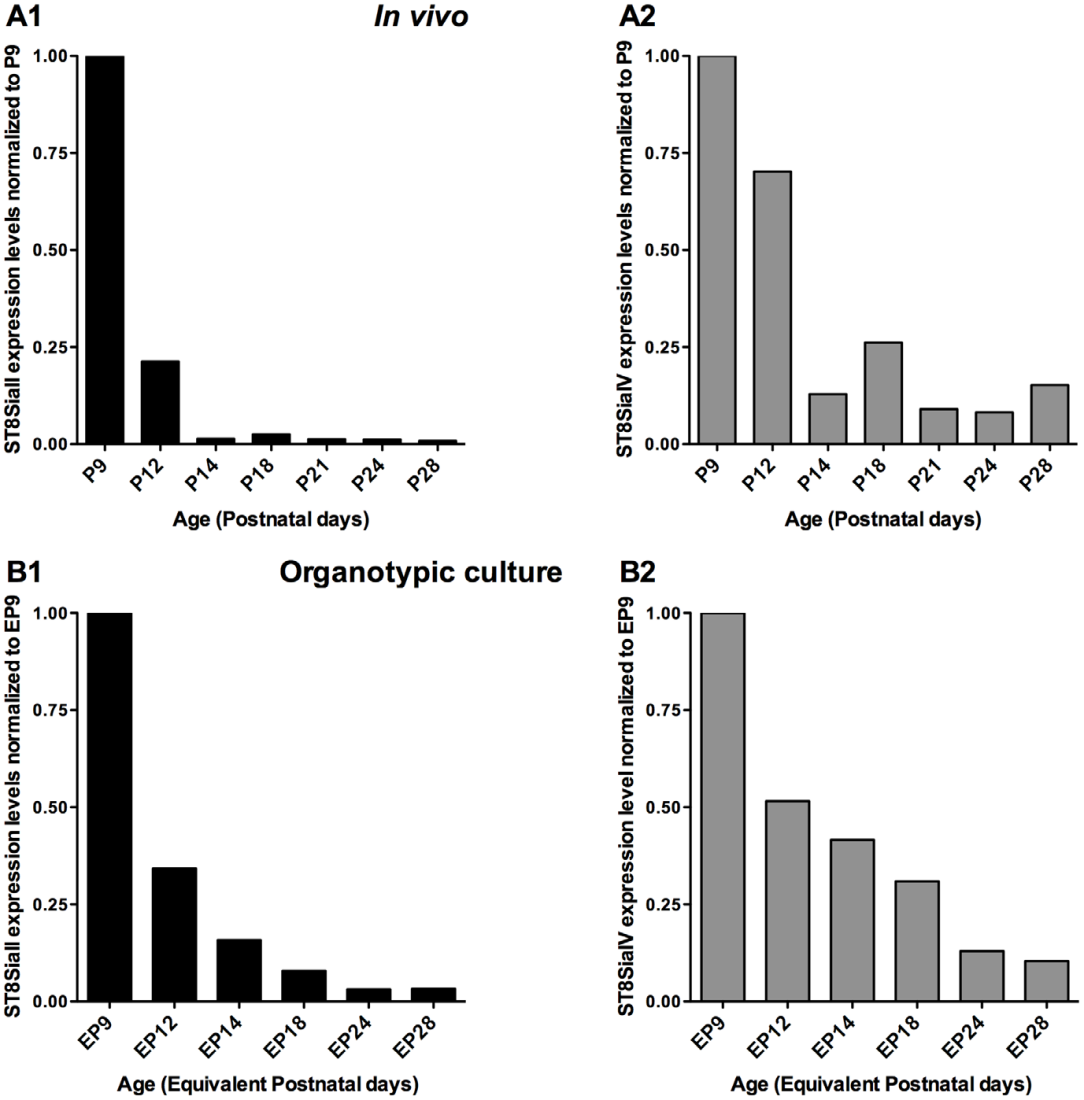
Expression levels of ST8SiaII (black bars) and ST8SiaIV (gray bars) mRNA decline in mouse visual cortex (A) and organotypic culture (B) with a similar time course during postnatal development. ST8SiaII and ST8SiaIV mRNA expression were first normalized to the internal control GAPDH. Bar graph represent the mean of at least two animals (A) or 6 cortical organotypic slices (B), normalized to the value obtained at P9.

We first characterized the time course of ST8SiaII and ST8SiaIV expression in mouse visual cortex (Figure 1A) and in organotypic culture prepared from mouse occipital cortex (Figure 1B) by using quantitative real-time PCR (qPCR) analysis. ST8SiaII expression sharply declined around eye opening (postnatal day (P) 13) and was nearly absent in mouse visual cortex from P14 through adulthood (Figure 1A1). ST8SiaIV expression levels were also diminished by P14, but less dramatically than its counterpart ST8SiaII; indeed low levels of PST transcripts could still be detected in adults (Figure 1A2). Overall, ST8SiaIV mRNA levels remained higher that ST8SiaII mRNA levels (Figure S1). The developmental decline in ST8SiaII and ST8SiaIV mRNA levels parallels the observed decline of PSA expression [8], thus suggesting that the limiting step regulating PSA expression levels is its synthesis.

**Figure 2:**
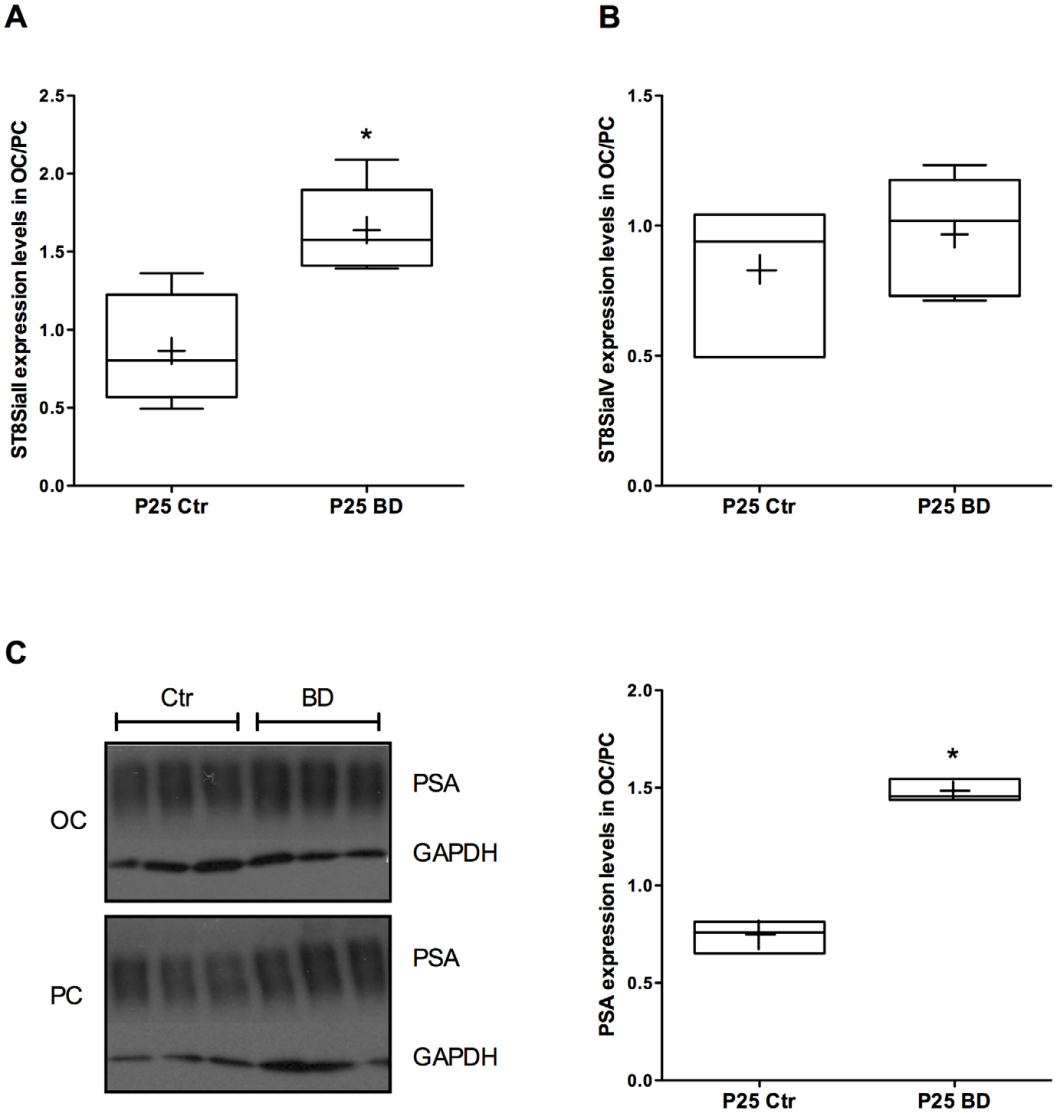
Sensory experience regulates ST8SiaII, but not ST8SiaIV, mRNA levels *in vivo*. (A–B) ST8SiaII (A), but not ST8SiaIV (B), mRNA levels are significantly higher in visual cortex from mice binocularly deprived (BD) from P13-25 (Mann Whitney-test, *p<0.01; n = 4 for Controls and n = 5 for BD animals). For each animal, ST8SiaII and ST8SiaIV expression levels in visual cortex (OC) are normalized to those found in parietal cortex (PC). (C) Left, immunoblot analysis of mouse visual cortex (OC) and parietal cortex (PC) using anti-PSA and anti-GAPDH antibodies. Right, quantification of PSA expression levels in mouse visual cortex (OC) normalized to parietal cortex (PC). PSA developmental down-regulation is impaired in binocular deprived animals (Mann-Whitney -test, *p<0.01; n = 3 for both Controls and BD animals). The cross in the graph represents the average, while the horizontal bar is the median value. The box shows the interquartile range, which is used as a measure of data spread - spanning 50% of a data set and eliminating the influence of outliers. The whiskers go down to the smallest value and up to the largest. When n = 3 or lower, there is no whiskers and the floating bar represents the maximum and the minimum values.

Previous data showed that PSA expression decreases after eye opening and that its decline is dependent on visual experience [8]. Whether ST8SiaII, ST8SiaIV or both are sensitive to visual experience is unknown. To assess the effect of visually-induced neuronal activity on ST8SiaII and ST8SiaIV gene expression, we binocularly deprived (BD) mice by eyelid suture from P13 to P25 and quantified ST8SiaII and ST8SiaIV transcript levels by qPCR. To correct for inter-individual variability in gene expression, we normalized the mRNA levels measured in occipital, visual cortex (OC) by those measured in parietal cortex (PC), as described in Di Cristo et al (2007) [8]. ST8SiaII, but not ST8SiaIV, mRNA levels were significantly higher in BD mice compared with non-deprived, age-matched, control mice (Figure 2A,B; n = 4 P25 Ctr mice and n = 5 P25 BD mice; Mann-Whitney test, p<0.01 for ST8SiaII, p>0.05 for ST8SiaIV). We further confirmed that PSA expression, quantified by immunoblot analysis, remained significantly higher in visual cortex from BD mice compared to P25 control littermates (Figure 2C, n = 3 P25 Ctr mice, n = 3 P25 BD mice; Mann-Whitney test, p<0.001), similarly to what occurs in mice dark-reared from birth [8]. Altogether, these results suggest that 1) ST8SiaII, but not ST8SiaIV, gene expression is regulated by visual experience, and 2) ST8SiaII is the main enzyme responsible for experience-dependent downregulation of PSA expression in the postnatal cortex around eye opening.

**Figure 3:**
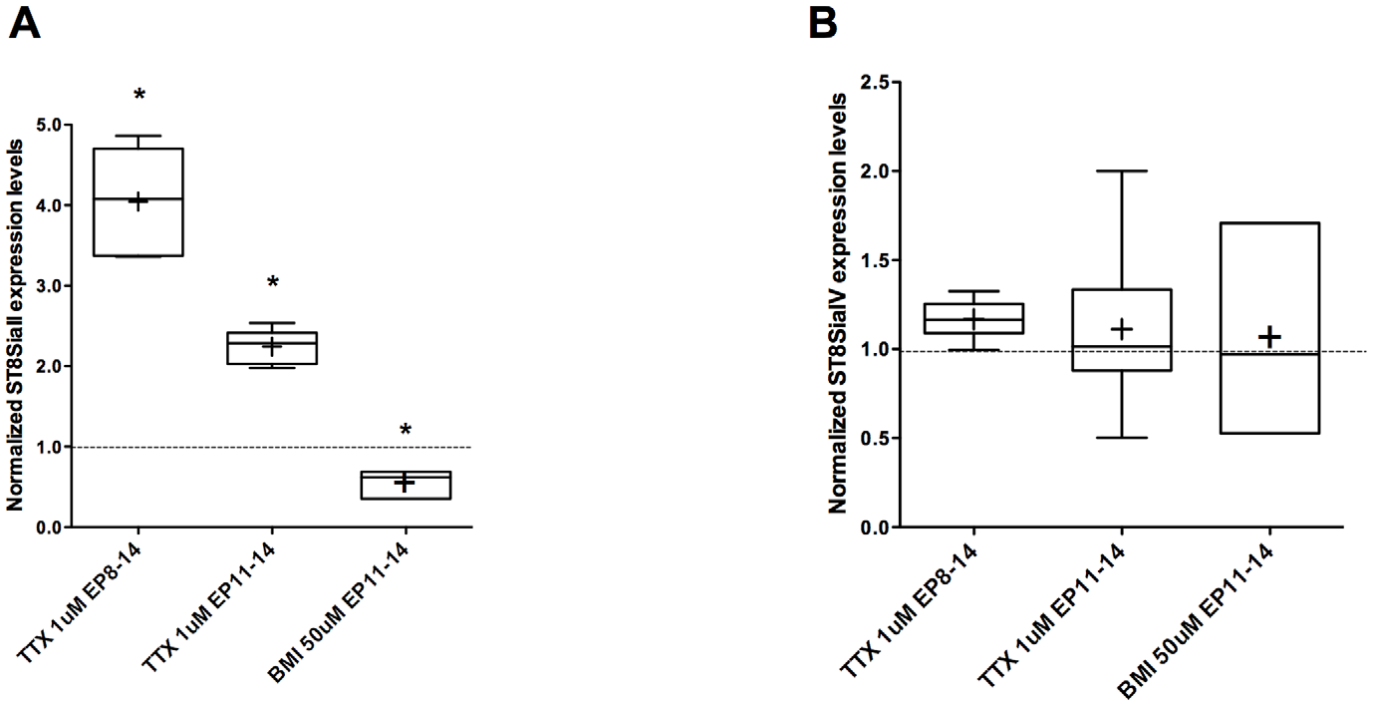
Spiking activity regulates ST8SiaII, but not ST8SiaIV, mRNA expression levels in cortical organotypic cultures. ST8SiaII (A) but not ST8SiaIV (B), developmental downregulation is impaired by TTX application (Two- Way ANOVA, Dunn's test p<0.05 for ST8SiaII in culture treated with TTX from EP8-14 and EP11-14; p>0.05 for ST8SiaIV). Conversely, increasing spiking activity levels by BMI treatment accelerates ST8SiaII (A; Two-Way ANOVA, Dunn's test p<0.05), but not ST8SiaIV (B; p>0.05) mRNA level downregulation. ST8SiaII and ST8SiaIV of each sample are first normalized to GAPDH levels, then to age-matched controls from the same litters and experiment. Graphs are plotted as explained in Figure 2.

To further examine the molecular mechanisms regulating activity-dependent ST8SiaII expression, we turned to cortical organotypic cultures. Many developmental processes occur in organotypic cultures with a time course similar to the *in vivo* brain [21], [22], [23]. In particular, PSA expression decreases between Equivalent Postnatal day 14 (EP 14 = P4+10 days *in vitro*) and EP24 in cortical organotypic cultures. *In vitro* studies revealed a similar developmental time course for ST8SiaII and ST8SiaIV expression, with both ST8SiaII and ST8SiaIV steadily declining after the first week *in vitro* (Figure 1B); the most important decline being observed by EP14. We further examined whether ST8SiaII gene expression was dependent on neuronal activity levels. To block spiking activity, 1 µM tetrodotoxin (TTX) was added to the culture media from EP8-14 (Figure 3). This time window was chosen because ST8SiaII expression sharply declined between EP8 and EP12 (Figure 1B1). We first observed that ST8SiaII and ST8SiaIV mRNA levels were very consistent within a single litter, but could vary a lot between litters. For this reason, each experiment was repeated using at least two different litters and raw data (ST8SiaII/GAPDH and ST8SiaIV/GAPDH) for both control and treatment groups were compared using 2-way anova, to account for both the treatment and the litter. To render the data graphically, we then plotted the ratio of ST8SiaII expression levels in treated over untreated controls for each experiment. We found that ST8SiaII, but not ST8SiaIV, developmental down-regulation was prevented by TTX application (Figure 3A, n = 6 TTX-treated samples from EP8-14; n = 6 Ctr samples; 2-way anova, post-hoc Dunn's test, p<0.05 for ST8SiaII, p>0.05 for ST8SiaIV). This effect was reproducible and independent from the litter. We next asked whether shorter period of spiking activity blockade was sufficient to affect ST8SiaII expression. ST8SiaII mRNA levels in TTX-treated cultures from EP11-14 were significantly increased compared to ST8SiaII levels in age-matched cultures (Figure 3A; n = 8 TTX-treated samples from EP11-14, n = 11 Ctr samples; 2-way anova, post-hoc Dunn's test, p<0.05). ST8SiaIV levels were not affected (Figure 3B; 2-way anova, post-hoc Dunn's test, p>0.05). ST8SiaII mRNA levels were proportional to the period of neuronal spiking blockade, indeed in cultures treated with TTX for 6-days we detected a 4-fold increase of ST8SiaII transcript vs 2.2-fold increase in cultures treated for 3 days. These changes are likely not due to tissue damage caused by prolonged blockade of spiking activity, as no neuronal death was reported following a 4-days TTX treatment [23].

Next we investigated whether increasing neuronal activity could accelerate the down-regulation of ST8SiaII mRNA. Treatment with 50 µM bicuculline methiodide (BMI), a GABA-A receptor inhibitor, in culture media from EP11-14 accelerated ST8SiaII mRNA reduction compared to aged-matched controls. No changes were observed for ST8SiaIV (Figure 3, n = 3 BMI-treated samples from EP11-14; n = 6 Ctr samples). Altogether, these data strongly support the hypothesis that ST8SiaII, but not ST8SiaIV, mRNA expression is dependent on neuronal activity levels during post-natal development in the cortex.

**Figure 4:**
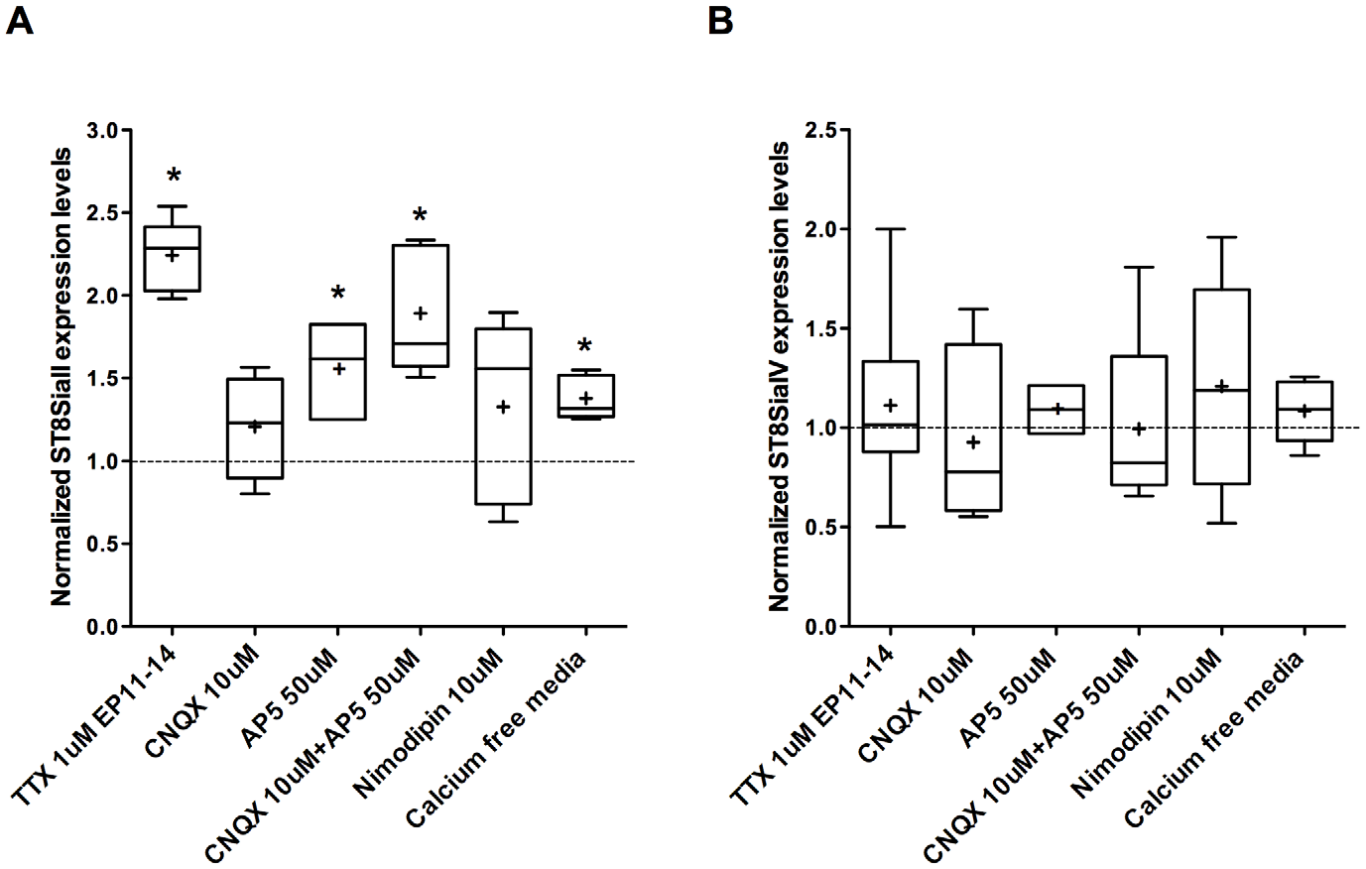
NMDA-mediated excitation regulates ST8SiaII mRNA expression levels, in part through Ca^2+^ dependent mechanisms. (A) ST8SiaII mRNA levels are significantly higher in organotypic cultures treated from EP11-14 with AP5, an inhibitor of NMDA receptor activation, compared to control values. This effect is potentiated by the simultaneous blockade of NMDA and AMPA receptor, via CNQX+AP5 treatment (Two-Way ANOVA, by post hoc Dunn's test, *p<0.05). Incubating organotypic cultures with Ca^2+^ free media causes a small but significant increase in STX expression levels compare to controls (*p<0.05). Blockade of L-type VGCC via nimodipine application does not significantly affect ST8SiaII mRNA levels (p>0.05). (B) None of these treatments affects ST8SiaIV mRNA levels (p>0.05). Graphs are plotted as explained in Figure 2.

To investigate whether glutamatergic neurotransmission was involved in ST8SiaII expression regulation, we blocked AMPA and NMDA receptors using respectively CNQX (10 µM) and AP5 (50 µM) from EP11-14. Compared to controls, ST8SiaII expression levels were significantly increased by NMDA but not AMPA receptor blockers alone, and by the combination of both (Figure 4A; n = 3 AP5-treated samples, n = 4 CNQX-treated samples, n = 5 CNQX+AP5 -treated samples, n = 6 Ctr samples; 2-way anova, post-hoc Dunn's test, after allowing for the effects of differences in litters, we obtained p<0.05 for AP5 and AP5+CNQX, p>0.05 for CNQX). Importantly, ST8SiaII levels in AP5 and AP5+CNQX-treated cultures were not significantly different from the one in TTX-treated cultures (1-way anova on ratios of STX expression levels in treated over untreated controls, post-hoc Dunn's test, p>0.05 for TTX treated culture vs AP5+CNQX and for TTX vs AP5; p<0.05 for TTX treated culture vs CNQX). The larger effect elicited by AP5 compared to CNQX application suggests that Ca^2+^ entry through NMDA receptor may play a role in the regulation of ST8SiaII expression. Spiking activity stimulates Ca^2+^ influx into the postsynaptic neuron through NMDA receptor and voltage-gated Ca^2+^ channel (VGCC); in turn Ca^2+^ triggers several signalling cascade leading directly to changes in gene expression [24], [25]. To investigate whether calcium entry through VGCC plays also a role in regulating ST8SiaII expression, we blocked VGCC using nimodipine (10 µM) from EP11-14. Contrary to NMDA blockade, the effect of nimodipine on ST8SiaII levels was very variable and on the whole, not significantly different from controls (Figure 4A, n = 5 nimodipine-treated samples, n = 7 Ctr samples; 2-way anova, post-hoc Dunn's test, p>0.05). To confirm that Ca^2+^ influx plays a role in the regulation of ST8SiaII expression, we incubated cortical cultures in calcium-free media from EP11-14. We found that ST8SiaII levels were slightly, but significantly, increased in this condition compared to controls (Figure 4A, n = 6 treated samples, n = 2 Ctr samples; t-test, p<0.05). Therefore, Ca^2+^ influx is at least one of the factor modulating ST8SiaII expression. ST8SiaIV mRNA levels were not significantly affected by any of these treatments (Figure 4B, 2-way anova, p>0.05). Taken together, these results suggest that the developmental decline of ST8SiaII, but not ST8SiaIV, mRNA levels during postnatal development in the cortex is mediated by NMDA receptor activation.

**Figure 5:**
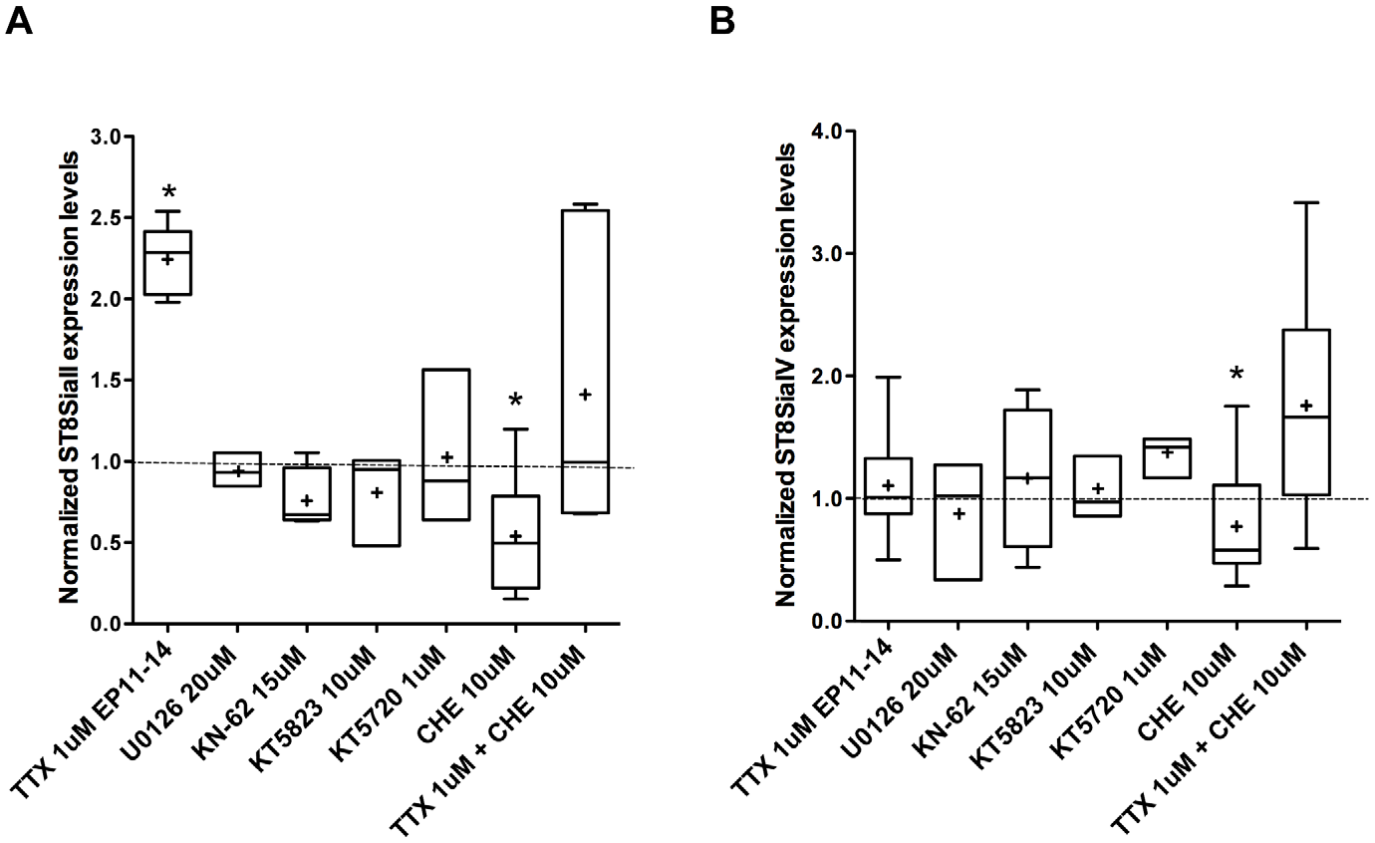
PKC signaling pathway positively regulates ST8SiaII and ST8SiaIV mRNA expression levels. Graphs illustrating the effect of the pharmacological inhibition of different signaling pathways on ST8SiaII (A) and ST8SiaIV (B) mRNA levels. Both ST8SiaII and ST8SiaIV mRNA levels are prematurely down-regulated in chelerythryne (CHE)-treated samples, compared to controls (Two-Way ANOVA, by post hoc Dunn's test, *p<0.05). TTX-induced increase in ST8SiaII mRNA levels (*p<0.05) does not occur in presence of CHE. Indeed, ST8SiaII levels in CHE+TTX cultures are not significantly different from those found in controls (Two-Way ANOVA, by post hoc Dunn's test, p>0.05). Neither ST8SiaII (A) or ST8SiaIV (A) mRNA levels are affected by the application of U0126, KN-62, KT5823 and KT5720 (Two-Way ANOVA, by post hoc Dunn's test, p>0.05). Graphs are plotted as explained in Figure 2.

Next, we tried to uncover which signaling pathways are involved in the activity-dependent downregulation of ST8SiaII transcripts in the postnatal cortex. NMDA receptor activation is known to activate many kinases, including: ERK [26], CAMKII [27], PKA [28] and PKG [29]. To test whether any of these pathways was involved in the regulation of ST8SiaII gene expression, we added to the culture media specific pharmacological inhibitors from EP11-14. In particular, we tested the effect of inhibitor of ERK (U0126, 20 µM), CaMKII (KN-62, 15 µM), PKA (KT5720, 1 µM) and PKG (KT5823, 10 µM). No significant difference in ST8SiaII and ST8SiaIV expression levels were reproducibly observed following any of these treatments (Figure 5, n = 3 U0126-treated samples, n = 4 KN-62-treated samples, n = 3 KT5720-treated samples, n = 3 KT5823- treated samples; two-way anova, p>0.05), therefore none of these kinases play a determinant role in regulating ST8SiaII or ST8SiaIV expression in the postnatal cortex.

*In vitro* studies suggest that ST8SiaII gene expression may be dependent on CREB or a CREB-like transcription factor [30]. PKC-mediated CREB activation could thus be involved in ST8SiaII transcription. Blocking PKC activity by adding 10 µM chelerythrine in the culture media from EP11-14 caused a reduction of both ST8SiaII and ST8SiaIV mRNA levels (Figure 5; n = 6 chelerythrine- chloride treated samples; n = 4 Ctr samples; 2-way anova post-hoc Dunn's test, p<0.05, after allowing for the effects of differences in litters). To test whether PKC activation modulates activity-dependent regulation of ST8SiaII mRNA levels, we treated organotypic cultures with 1 µM TTX+10 µM chelerythrine from EP11-14. ST8SiaII mRNA levels in the treated group were not significantly different from untreated controls (Figure 5A, n = 4 treated samples, n = 4 Ctr samples; two-way anova, p>0.05), suggesting that PKC activation is at least partially involved in maintaining high ST8SiaII mRNA levels when neuronal spiking activity is blocked. ST8SiaIV mRNA levels were very variable in the treated samples, increasing in some samples and decreasing in others (Figure 5B, 2-way anova, p>0.05).

Altogether, these results suggest that ST8SiaII mRNA levels are finely tuned by NMDA-mediated excitation and PKC activation during postnatal development in visual cortex.

## Discussion

Previous work has shown that during development, PSA expression is dependent on sensory experience in mouse visual cortex, and its downregulation regulates the timing for GABAergic synapse maturation and the onset of ocular dominance plasticity [8]. The cellular mechanism that couples neural activity and PSA expression is a key to linking this general modulator of cell interaction to specific neural developmental events. The purpose of this study was to identify cellular mechanisms by which the experience-dependent regulation of PSA occurs, and to identify signaling pathways that might couple visual activity with PSA biosynthetic machinery.

We analyzed the developmental time course and the effect of visual deprivation in vivo on the expression of the genes encoding the enzymes responsible for PSA synthesis in mouse brain, the polysialyltransferases ST8SiaII and ST8SiaIV. The results obtained were similar for ST8SiaII and ST8SiaIV, in that the disappearance of NCAM polysialylation previously found during the period of synaptogenesis [8] is accompanied by a corresponding decrease in the levels of both ST8SiaII and ST8SiaIV transcripts. Similar results were found in organotypic cultures system where PSA levels [8], ST8SiaII and ST8SiaIV mRNA levels declined over the same period, in parallel to a steady increase in the levels of spontaneous activity [31], [32]. Remarkably, mRNA levels for ST8SiaII, but not ST8SiaIV, remain higher in the visual cortex of binocular deprived mice as compared to control littermates and in TTX-treated organotypic cultures, paralleling the increase in PSA expression levels [8]. These findings suggest that activity-dependent regulation of ST8SiaII transcript levels may be the molecular mechanism linking sensory experience and PSA expression in postnatal visual cortex.

This findings are consistent with previous studies with whole brain extracts, which showed that the developmental downregulation of PSA was preceded by decreased levels of polysialyltransferase transcripts and accompanied by a shift in the ratios between St8SiaII and IV [33]. Moreover, the analysis of mice deficient for either St8SiaII or ST8SiaIV suggests a predominant role of ST8SiaII in PSA biosynthesis on NCAM at early postnatal stages [33]. Here, we showed that visual experience is at least one of the factors regulating ST8SiaII downregulation. We further showed that the increase in intracortical network activity occurring around EP8-12 [31], [32] is sufficient to trigger ST8SiaII decrease *in vitro*. Neuronal activity in cultured cortical slices seems to mature at a similar rate to that *in vivo*. Indeed, it has been reported that spontaneous firing is extremely rare in P7 cortical slices but increases at later stages to resemble the pattern of activity observed in the adult [34]. Moreover, in freely moving rats, firing rates in the cortex increase around P10 [35]. These similarities in developmental timing of spontaneous activity between the intact and cultured cortex suggest that intrinsic electrical properties such as membrane properties and the balance in the formation of inhibitory and excitatory synapses is preserved in cultured cortical slices. Network activity likely contributes to several aspects of maturation of the visual cortex, including eye specific segregation and intracortical axon branching [32], [36], which occurs before eye opening in rodent or birth in human. The onset of visual inputs would then contribute to the refinement and maintenance of specific connections necessary for the development of mature visual responses. In this context, it is possible that STX downregulation *in vivo* is triggered by the increase in network activity and is modulated and maintained by visual inputs after eye opening. It will be interesting to investigate which are the environmental signals that promote this switch in other cortical regions.

PSA is widely expressed throughout the visual cortex during the first 3 postnatal weeks [8]; however it is currently unknown whether it is preferentially localized to particular cellular surfaces, such as GABAergic interneurons, excitatory pyramidal neurons or glia cells, or subcellular compartments, i.g. axons, somata or dendrites. In contrast, in the adult cortex, PSA expression is restricted mainly to interneurons in the medial prefrontal cortex (mPFC, [37], [38]). Dopamine acting on D2 receptors [37] and the antidepressant fluoxetine acting through 5-HT3 receptors [38] have been shown to modulate PSA expression levels in the adult mPFC, suggesting that neuromodulator-mediated changes in PSA expression might promote structural plasticity of the adult mPFC. These changes in PSA expression are likely mediated by ST8SiaIV, as ST8SiaII is reduced to almost undetectable levels in the adult cortex.

Our evidence suggests that activity-dependent reduction in ST8SiaII transcription required (1) activation of NMDA receptors, and (2) calcium entry into the cell likely through the NMDA receptor. Under physiological conditions, extracellular Mg^2+^ blocks the NMDA receptor channel at the resting membrane potential. High-frequency stimulation of presynaptic afferents should activate non-NMDA receptor channels sufficiently to depolarize the postsynaptic cell, remove the Mg^2+^ blockade, and permit Ca^2+^ entry via NMDA receptor channels. In organotypic cultures, the developmental decrease of ST8SiaII mRNA levels was suppressed by AP5, but not by the voltage-gated Ca^2+^ channel blocker Nimopidin, suggesting a negligible role in this effect for Ca^2+^ influx through voltage-gated Ca^2+^ channels. While ST8SiaII levels were significantly increased in Ca^2+^ free-medium treated cultures as compared to controls, there were also significantly lower than in AP5-treated cultures, suggesting that additional NMDA-mediated mechanisms, other than Ca^2+^ influx, may modulate ST8SiaII expression.

NMDA receptor activation has been shown to differentially regulate PSA expression in several systems, with the direction of the change depending from the age and the region [39]. Similarly, the differential effect of NMDA activation on ST8SiaII and ST8SiaIV transcript levels might be age specific in the visual cortex.

In addition to transcriptional control of polysialyltransferase(s), PSA expression at the cell surface could also be affected by nontranscriptional modulation of either ST8SiaII or ST8SiaIV activity or both. For example, it has been suggested that calcium-dependent regulatory mechanism [40] and polysialyltransferase phosphorylation [41] may be involved in the regulation of enzymatic activity. Finally, PSA expression on the cell surface can be more locally and rapidly regulated by activity-dependent differential delivery of presynthesized PSA–NCAM to the cell surface. Indeed, intracellular granules containing PSA have been described in cortical neurons and the calcium-dependent fusion of these granules with the plasma membrane can be induced by depolarization of the membrane [42]. Further, in oligodendrocyte precursors, NMDA can induce a glutamate receptor-dependent influx of calcium that is thought to enhance transport of PSA–NCAM to the cell surface [43].

The molecular mechanisms responsible for the activity-dependent reduction of ST8SiaII transcripts are still unknown. ST8SiaII transcription could be modulated by a transcriptional repressor, such as repressor-element 1 silencing transcription factor (REST) [44]. On the other hand, ST8SiaII transcripts levels could also be negatively regulated by micro-RNA (mi-R). Indeed, miRs have been shown to play a role in multiple developemental processes, including synapse formation and plasticity [45]; moreover, the expression of some miRs can be modulated by neuronal activity [46], [47]. Further experiments will be required to investigate whether specific miR could target ST8SiaII mRNA and reduce its stability.

Our findings suggest that both ST8SiaII and ST8SiaIV gene expression is regulated by PKC; indeed treating organotypic cultures with chelerythryne, an inhibitor of all PKC isoforms, induces a precocious reduction of both ST8SiaII and ST8SiaIV levels. Moreover, inhibition of PKC impairs the TTX-induced increase of ST8SiaII transcripts. PKC-mediated CREB activation could be involved in ST8SiaII and ST8SiaIV gene transcription. In vitro studies indicate that ST8SiaII, (but not ST8SiaIV) gene expression is dependent on CREB or a CREB-like transcription factor [30]. In addition, it has been suggested that PKC can phosphorylate a repressor complex formed by HDAC1/2, thereby allowing gene transcription [48]. Further experiments will be required to establish whether the HDAC1/2 complex is involved in ST8SiaII/ST8SiaIV expression. Interestingly, Gallagher et al (2001) [41] demonstrated that protein kinase C delta (PKCδ) negatively regulates polysialyltransferase activity in the rat hippocampus during memory consolidation. Thus, it is possible that different PKC isoforms play different roles or, alternatively, their effects could be age- or brain region- specific. Overall, the findings regarding the biosynthesis of ST8SiaII and ST8SiaIV are remarkable with respect to the specificity of the pharmacology: indeed, no perturbation of major second messenger pathways other than PKC pathway had any effect of ST8SiaII and ST8SiaIV synthesis, indicating that polysialylation is not easily perturbed by a wide spectrum of factors.

In conclusion, our findings suggest a dynamic model, where ST8SiaII mRNA levels are positively regulated by PKC-mediated signaling and decreased by neural activity-dependent mechanisms, in the developing visual cortex. The balance of these processes controls polysialyltransferases activity and, consequently, PSA expression levels. PSA levels, in turn, regulate the timing of specific cell-cell interaction events leading to neuronal circuit formation and plasticity. Contrary to ST8SiaII, ST8SiaIV mRNA levels are not influenced by neuronal activity or any other of the studied kinases (ERK, PKG, CaMKII, PKA) during the first weeks of postnatal development. It is however possible that ST8SiaIV might respond to extracellular clues, and thus contribute to changes in PSA expression, in adults or/and in different brain regions (for example in mPFC [37], [38].

The signaling mechanisms regulating PSA synthesis are of prime importance for neuronal circuit formation and plasticity. In addition to homophilic NCAM binding [49], PSA regulates various adhesion and signaling molecules that play a role in synaptic plasticity, including cadherins, LICAMs, integrins [50], FGF receptors [51], and BDNF [52], [53]. Further, it has been recently shown that PSA modulates NMDA receptor signaling [54]. Considering the multifaceted aspects of PSA interactions, it is tempting to speculate that alterations of PSA regulation might be implicated in disorders of neurodevelopmental origin. Recently, genetic variations in ST8SiaII have been implicated in schizophrenia [55], [56]. In particular, Isomura and colleagues [57] showed that a single nucleotide polymorphisms (SNPs; STX(G421A)) of the coding region of ST8SiaII indentified in schizophrenic patients causes a dramatic decrease in PSA synthetic activity on NCAM and produces PSA with shorter chain length. Interestingly, the STX(G421A)-derived PSA-NCAM completely loses the dopamine binding activity and has greatly diminished BDNF binding activity, These findings suggest a potential mechanism by which genetic interference with the complex coordination of NCAM polysialylation may lead to a neurodevelopmental predisposition to psychiatric diseases. In addition, modulation of PSA production in the central nervous system may have therapeutic potential [13], [58], but an understanding of the key processes leading to the polysialylation of NCAM by ST8SiaII and ST8SiaIV is required before such treatment could be envisaged. The investigation of such mechanisms under normal and pathological conditions could pave the way to new treatment strategies to re-induce structural plasticity.

## Methods

### Animals

C57BL/6 mice were purchased from Harlan Laboratories (Montreal, QC, Canada). Full details of the study have been approved by the Canadian Council on Animal Care (CCAC) committee of CHU Ste-Justine Research Center (Approval ID: 427). All experiments were carried out in compliance with the ethical rules of CHU.Ste-Justine Research Center and Université de Montréal.

### Organotypic culture

Postnatal day 4 (P4) to P6 mouse pups were decapitated and brains were rapidly removed and immersed in ice-cold culture medium (MEM enriched with Glutamax (cat. no. 42360, Invitrogen, Carlsbad, CA, USA), 20% Horse serum, 1 mM glutamine, 2 mM MgSO_4_, 1 mM CaCl_2_, 0.0012% ascorbic acid, 1 µg/ml insulin, 30.04 mM Hepes, 5.24 mM NaHCO_3_, 12.88 mM D-glucose, pH 7.27). 400 µm thick coronal brain slices were obtained using a Chopper (Stoelting, Wood Dale, IL, USA). Slices (usually 2–3) were placed onto Millicell membrane inserts (cat. no. PICMORG50), in a 6-well plate containing 750 µl of culture medium per well. Tissues were incubated at 34°C in a 5% CO2-enriched atmosphere humidified incubator. Medium was changed every second day. In experiments in which conditioned media was used, pharmacological agents were mixed directly with the media, filtered using 0.22 µm filter fixed to a syringe, and added to cultures during specific time windows. Drugs were dissolved in DMSO and final concentration in culture media were as follow: Nimodipine 10 µM, K252a 40 µM, U0126 20 µM, KN-62 15 µM, KT5823 10 µM, KT5720 1 µM, and Chelerythrine chloride (CHE) 10 µM (Sigma-Aldrich, St-Louis, MO, USA). Bicuculline Methiodide (BMI, 50 µM, Fluka Chemical Corp, Ronkonkoma, NY, USA) was dissolved in deionized water. CNQX (10 µM, Tocris Bioscience) was dissolved in HCl 1N. AP5 (50 µM, Tocris Bioscience) was dissolved in NaOH 1 M. Finally, Tetrodotoxin (TTX, 1 µM, Alomone Labs, Jerusalem, Israel), was diluted in citrate buffer pH 4.8. For experiments requiring calcium-free media, culture media was essentially the same as described above, with the exception that MEM enriched with Glutamax was replaced by S-MEM (Invitrogen, cat. no 11380-037) and no CaCl_2_ was added.

### Binocular deprivation

To assess the effect of visual deprivation on gene expression, P13 mice were anesthetized by isoflurane (2%) and binocularly deprived. Procedures were performed prior to eye opening. Lid margin were trimmed and sutured with 5-0 Monosoft Nylon. Mice recovered rapidly after the cessation of anesthesia and were returned to their cages. Mice showing lid opening were not included in the experiments. Mice were sacrificed at P25 by decapitation. Visual cortices from both hemispheres were collected and either frozen in liquid nitrogen and stored at −80°C if used for protein extraction or kept at 4°C in RNAlater solution (Qiagen Inc, Mississauga, ON, Canada) if used for RNA extraction.

### Immunoblotting

Immunoblotting was performed essentially as described [8]. Protein lysates were prepared by homogenizing tissue from either mouse visual cortex or cortical organotypic culture in 50 mM Tris-HCl (pH 7.6), 150 mM NaCl, 2 mM EDTA, 1% Igepal CA-630, and 1× protease inhibitor cocktail (Roche, Basel, Switzerland). For *in vitro* analysis, 4–6 cortical slices were collected and pooled together for each developmental time point or pharmacological experiment; one mouse for each age point and at least 3 mice for sensory deprived experiments were used for *in vivo* analysis. Tissues were disrupted by pipetting up & down. The amount of protein extracted was quantified by Bradford protein assay (cat. no. 500-0006, Bio-Rad Laboratories, Hercules, CA, USA), and samples were adjusted to equivalent concentrations with deionized water. Samples were mixed with an equal volume of 2× Laemmli buffer, boiled for 5 min, and stored at −80°C. Equal amount were loaded to each lane. Proteins were separated using 6.5% polyacrylamide separation gels with 5% stacking gels (Bio-Rad Laboratories). Transfer process occurred onto Millipore Immobilon-P polyvinylidene fluoride (PVDF) membrane (Millipore, Billerica, MA, USA), which was then blocked by incubation in TBS-T (0.1% Tween-20) with 5% dried milk. Membranes were probed with anti-PSA (1∶1000, mouse monoclonal IgM, cat no. MAB5324, Chemicon (now part of Millipore) and anti-GAPDH (1∶4000, mouse monoclonal IgG, cat no. AB4300, Applied Biosystems, Foster City, CA, USA). Horseradish peroxidase-conjugated anti-mouse secondary antibody (Invitrogen) (Amersham - acquired by GE healthcare Bio-Sciences Corp, Piscataway, NY, USA) was added to the blots prior to immunoreactive band detection by Western Lighting Plus Chemiluminescence Reagent (cat no. NEL103, Perkin-Elmer, Waltham, MA, USA). Signals were visualized by exposing membrane to Bioflex autoradiography film (InterScience, Markham, ON, Canada). Normalization of the amount of PSA to GAPDH was performed by scanning non-saturated immunoblots and analyzed band intensities with Image J software.

### Quantitative Real-Time PCR

One mouse for each age point and at least 3 mice for binocular deprivation experiments were used for *in vivo* analysis. For *in vitro* analysis, 4–6 cortical slices were collected and pooled together for each developmental age point or pharmacological treatment. All samples were kept at 4°C in RNAlater solution (Qiagen Inc) for no more than one month prior to RNA extraction. RNA was extracted using the RNeasy mini kit (cat no. 74104, Qiagen Inc) following the manufacturer's instructions. RNA quantity and purity were determined by measuring the absorbance at 260 and 280 nm. Samples were stored at −80°C. Reverse transcriptase reaction for cDNA synthesis was performed using RT Omniscript kit (cat no. 205111, Qiagen Inc). RNA was first denatured at 65°C for 5 min. Each 20 µl-reaction contained 1 µg of RNA, 1× RT Omniscript reaction buffer, 0.5 mM of each dNTP, 4 U RT Omniscript enzyme, 1 µM Oligo-dT primers (Invitrogen), and 10 U RNase inhibitor (Invitrogen). Samples were incubated at 37°C for 1 h, and RT enzyme was inactivated at 93°C for 5 min. Experiments were carried-out on an ABI Prism 7900 Sequence Detection System (Applied Biosystems) using 384-wells plate. Each 10 µl reaction contained 1.5 µl of a 1/10 dilution of the cDNA reaction, 1× SYBR Green PCR Master Mix (cat no. 4309155, Applied Biosystems) and 0.4 nM exon-spanning primers (ST8SiaII 5′-CTCTCTGAGGATCAGGAAGCAAA-3′ and 5′-AATAATGTCTCCAGGCTTCAGGG-3′, ST8SiaIV 5′-GGAGATGGTGAACTGTGTTTGAG-3′ and 5′-ACAGAATGTTGGAAGATGGTGGAG-3′, GAPDH 5′-GGTCGGTGTGAACGGATTTGGC-3′ and 5′-TTGCCGTGAGTGGAGTCATACTGG-3′). Cycling conditions included a 10 min initial denaturation step at 95°C, followed by 42 cycles at 95°C for 15 sec and 60°C for 1 min. Melting curve analysis consisted of one cycle at 95°C for 15 sec, followed by 15 sec at 60°C, and 95°C for 15 sec. Note that all developmental time courses were normalized using both GAPDH and β-actin and results were identical. However, following experiments were performed using GAPDH only because it has been reported that β-actin expression might be regulated by neural activity.

### qPCR data analysis

Relative quantification of ST8SiaII, ST8SiaIV and GAPDH transcripts was determined by the standard curve method using serial dilution (1/2, 1/10, 1/50, 1/250, 1/1250) of a freshly prepared cDNA from P4 visual cortex RNA. Data was analyzed using the Real-Time PCR System Sequence Detection Software v2.2.2. Briefly, the amount of PCR transcripts was measured based on the threshold cycle (Ct), which is the cycle where fluorescence is detected above baseline level. Each dilution of the standard curve was performed in triplicate. Standard curves were obtained by plotting the mean Ct values against the log concentration of the input starting material. Standard curves were then used to obtain the amount of transcripts for each sample. Each sample was analyzed in triplicate for ST8SiaII, ST8SiaIV and GAPDH. The mean relative amount of transcripts of ST8SiaII and ST8SiaIV of each sample was normalized to GAPDH levels.

### Statistics

For *in vivo* experiment, we normalized mRNA or protein levels measured in occipital, visual cortex (OC) by the one measured in parietal cortex (PC), as described in Di Cristo et al (2007) [8], to correct for inter-individual variability in gene expression. PSA protein and ST8SiaII and ST8SiaIV mRNA normalized values obtained in controls and binocular deprived animals were compared using Mann-Whitney test on ranks.

For *in vitro* experiment, ST8SiaII and ST8SiaIV mRNA levels were very consistent within a single litter, but could vary a lot between litters. For this reason, each experiment was repeated using at least two different litters and raw data (ST8SiaII/GAPDH and ST8SiaIV\GAPDH) for both control and treatment groups were compared using 2-way anova, to account for both the treatment and the litter. Ratios treatment/controls were then compared between them Mann-Whitney test on ranks.

## Supporting Information

Figure S1 **Graphs showing mRNA expression levels of ST8SiaII and ST8SiaIV during postnatal development** ***in vivo*** **and** ***in vitro*** **.** Black circles represent ST8SiaII and black squares indicate ST8SiaIV mRNA expression levels. (**A**) ST8SiaII and ST8SiaIV transcript levels decline during development in visual cortex *in vivo*; ST8SiaIV expression remains higher than ST8SiaII from P9 through adulthood. (**B**) A similar expression pattern can be observed in cortical organotypic cultures. ST8SiaII and ST8SiaIV raw values are normalized to GAPDH.
(TIFF)
