# Supplementary figures and images for: Sensory Experience Differentially Modulates the mRNA Expression of the Polysialyltransferases ST8SiaII and ST8SiaIV in Postnatal Mouse Visual Cortex
Source: PLoS One. 2011 Sep 21;6(9):e24874. doi: 10.1371/journal.pone.0024874 (PMC3177851; doi:10.1371/journal.pone.0024874)

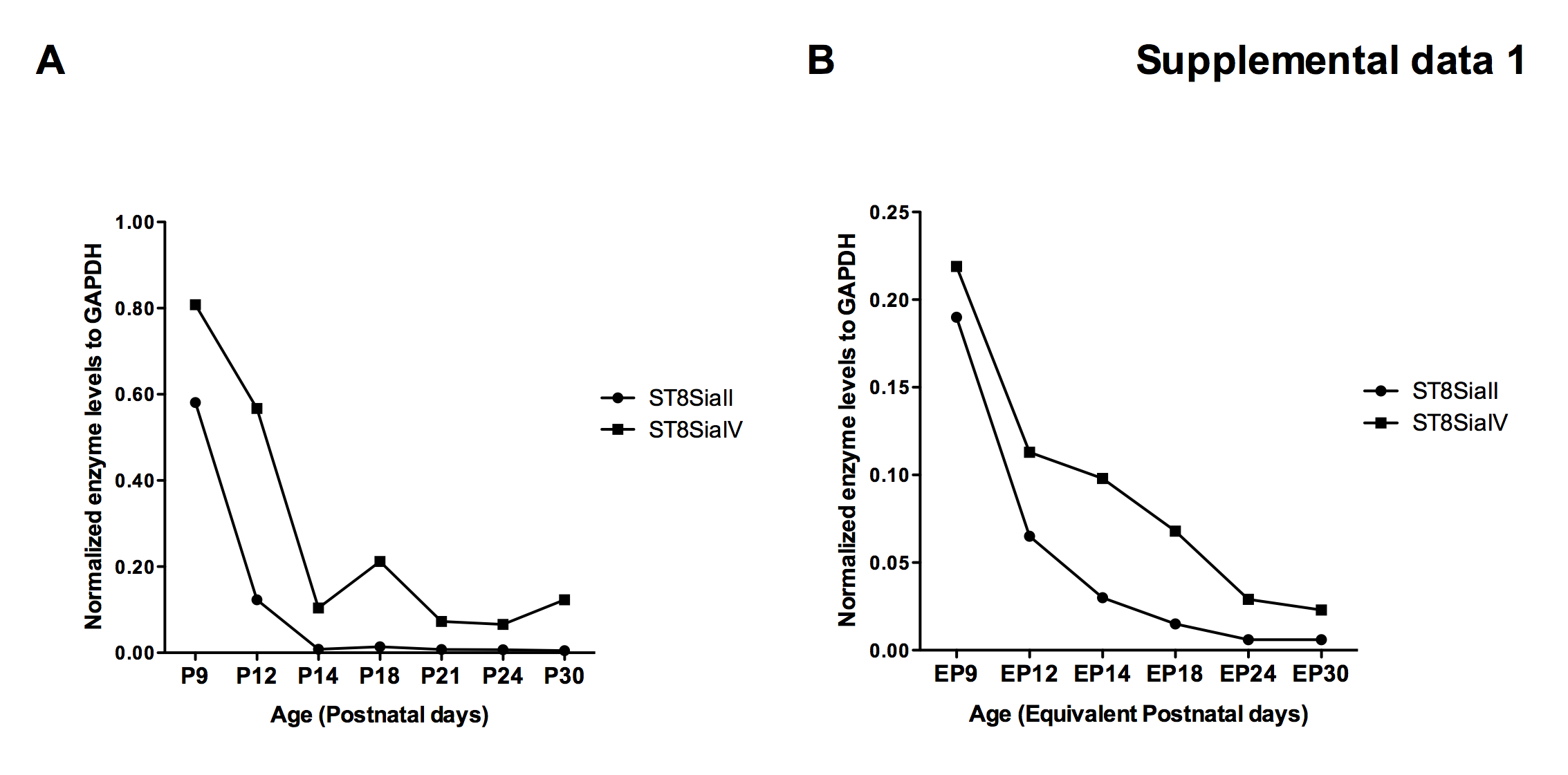

Supplement: Figure S1 — Graphs showing mRNA expression levels of ST8SiaII and ST8SiaIV during postnatal development in vivo and in vitro . Black circles represent ST8SiaII and black squares indicate ST8SiaIV mRNA expression levels. (A) ST8SiaII and ST8SiaIV transcript levels decline during development in visual cortex in vivo; ST8SiaIV expression remains higher than ST8SiaII from P9 through adulthood. (B) A similar expression pattern can be observed in cortical organotypic cultures. ST8SiaII and ST8SiaIV raw values are normalized to GAPDH. (TIFF) [file pone.0024874.s001.tiff]
